# Autophagy Promotes Duck Tembusu Virus Replication by Suppressing p62/SQSTM1-Mediated Innate Immune Responses In Vitro

**DOI:** 10.3390/vaccines8010022

**Published:** 2020-01-13

**Authors:** Zhiqiang Hu, Yuhong Pan, Anchun Cheng, Xingcui Zhang, Mingshu Wang, Shun Chen, Dekang Zhu, Mafeng Liu, Qiao Yang, Ying Wu, Xinxin Zhao, Juan Huang, Shaqiu Zhang, Sai Mao, Xumin Ou, Yanling Yu, Ling Zhang, Yunya Liu, Bin Tian, Leichang Pan, Mujeeb Ur Rehman, Zhongqiong Yin, Renyong Jia

**Affiliations:** 1 Research Center of Avian Disease, Sichuan Agricultural University, Chengdu 611130, China; zhiqianghu0624@163.com (Z.H.); 18227587189@163.com (Y.P.); chenganchun@vip.163.com (A.C.); zhangxc923@163.com (X.Z.); mshwang@163.com (M.W.); sophia_cs@163.com (S.C.); zdk24@sicau.edu.cn (D.Z.); liumafengra@163.com (M.L.); yangqiao721521@sina.com (Q.Y.); yingzi_no1@126.com (Y.W.); xxinzhao@sicau.edu.cn (X.Z.); huangjuan610@163.com (J.H.); shaqiu86@hotmail.com (S.Z.); sarrawin@163.com (S.M.); omlx19881130@163.com (X.O.); yanling3525@163.com (Y.Y.); zl97451@126.com (L.Z.); yunnyaaliu@163.com (Y.L.); btian_1985@163.com (B.T.); pl2007@126.com (L.P.); mujeebnasar@yahoo.com (M.U.R.); 2 Institute of Preventive Veterinary Medicine, Sichuan Agricultural University, Chengdu 611130, China; 3 Key Laboratory of Animal Disease and Human Health of Sichuan Province, Chengdu 611130, China; yinzhongq@163.com

**Keywords:** duck tembusu virus, autophagy, p62, p-TBK1, NF-κB, IRF7

## Abstract

Duck Tembusu virus (DTMUV) has recently appeared in ducks in China and the key cellular determiners for DTMUV replication in host cells remain unknown. Autophagy is an evolutionarily conserved cellular process that has been reported to facilitate flavivirus replication. In this study, we utilized primary duck embryo fibroblast (DEF) as the cell model and found that DTMUV infection triggered LC3-II increase and polyubiquitin-binding protein sequestosome 1 (p62) decrease, confirming that complete autophagy occurred in DEF cells. The induction of autophagy by pharmacological treatment increased DTMUV replication in DEF cells, whereas the inhibition of autophagy with pharmacological treatments or RNA interference decreased DTMUV replication. Inhibiting autophagy enhanced the activation of the nuclear factor kappa-light-chain-enhancer of activated B cells (NF-κB) and interferon regulatory factor 7 (IRF7) pathways and increased the p62 protein level in DTMUV-infected cells. We further found that the overexpression of p62 decreased DTMUV replication and inhibited the activation of the NF-κB and IRF7 pathways, and changes in the NF-κB and IRF7 pathways were consistent with the level of phosphorylated TANK-binding kinase 1 (p-TBK1). Opposite results were found in p62 knockdown cells. In summary, we found that autophagy-mediated p62 degradation acted as a new strategy for DTMUV to evade host innate immunity.

## 1. Introduction

Duck Tembusu virus (DTMUV) is a single-strand positive sense RNA virus that is classified as a member of the *Flavivirus genus* within the Flaviviridae family [1]. It was first reported in South East China in April 2010 and always caused a decline in egg production in laying ducks [1,2,3]. DTMUV infection has also been associated with viral encephalitis in young ducks [4] and mice [5], which is similar to the neurologic symptoms caused by other flavivirus members such as Zika virus (ZIKA) [6], West Nile virus (WNV) [7], and Japanese encephalitis virus (JEV) [8]. At present, the mechanism of DTMUV replication in host cells remains limited, and prevention with inactivated vaccines is the sole method of protection against DTMUV [9]. It is necessary to study the key cellular determinants for DTMUV replication to develop more efficient antiviral strategies and enrich our current understanding of flaviviruses.

Autophagy was initially discovered in yeast, and the process of autophagy is conserved in all eukaryotes including plants, yeast, and animals [10]. It is a lysosomal degradation pathway that generates double-membrane vesicles that deliver the cytoplasmic cargo to the lysosome [11]. Autophagy is monitored by the lipidation rate of microtubule-associated protein 1 light chain 3 (LC3) and the degradation of polyubiquitin-binding protein sequestosome 1 (SQSTM1/p62, and p62 hereafter). When autophagy occurs, LC3 will be converted from LC3-I to LC3-II with the phosphatidylethanolamine by E3-like conjugation enzymes, which are located on autophagosomes. However, the complete autophagy involves not only the enhancement of autophagosome biogenesis but also the trafficking to lysosomes. Autophagic flux, which refers to the entire process of autophagy, is a more accurate index to judge autophagic activity. The p62 protein serves as a link between LC3 and ubiquitinated substrates [12], which then become incorporated into the completed autophagosome and are degraded into autolysosomes, thus serving as an index of autophagic degradation [13].

Autophagy has been reported to act as a regulator of antiviral immune responses. For instance, the Atg5–Atg12 complex, an ubiquitin-conjugation system that is essential for the formation of preautophagosomes, positively regulates anti-viral NF-κB and IRF3 signaling in foot-and-mouth disease virus infection [14,15]. The Beclin 1-ubiquitin-specific protease 19 (BECN1–USP19) axis, a positive regulator of autophagy initiation and progression, plays a role in the crosstalk between autophagy and retinoic acid-inducible gene I (RIG-I) -mediated type I interferon signaling [16]. In addition, the autophagic cargo adaptor, p62, also plays multiple roles in modulating innate immune responses. For example, p62 has been reported to cause the degradation of RIG-I, a crucial member of the RIG-I-like receptors (RLRs) family, by its function as an autophagic cargo adaptor in selective autophagy [17]. Thaneas et al. also found that p62 was mediated with STING degradation by its ubiquitination to autophagosomes [18]. Its multiple roles in modulating innate immune responses may be caused by its multiple domains [19].

In this study, we might have found another pathway of autophagy regulating antiviral immunity. DTMUV-induced autophagy caused the degradation of p62, which regulated immune responses by enhancing TANK-binding kinase 1 (TBK1) phosphorylation and then affected the replication of DTMUV in duck embryo fibroblast (DEF) cells.

## 2. Materials and Methods

### 2.1. Antibodies and Chemicals

The primary antibodies of anti-LC3 (14600-1-AP), anti-Beclin 1 (11306-1-AP), anti-Flag (20543-1-AP), and anti-β-actin (60008-1-Ig) were purchased from Proteintech (Wuhan, China). Anti-SQSTM1/p62 (5114) was purchased from Cell Signaling Technology (Danvers, MA, USA) and anti-p-TBK1 (bs-3440R) was purchased from Bioss Antibodies (Beijing, China). The monoclonal antibody against E protein was custom-made by a biological company, and the immunogen was a recombinant E protein obtained from the DTMUV CQW1 E gene [20]. Horseradish peroxidases (HRP) conjugated to goat anti-rabbit (BF03008) or anti-mouse secondary antibodies (BF03001) were purchased from Beijing Biodragon Immunotechnologies (Beijing, China). Rapamycin (Rapa) (HY-10219), 3-methyladenine (3-MA) (HY-19312), chloroquine (CQ) (HY-17589), and aloxistatin (E64d) (HY-100229) were purchased from MedChemExpress (MCE, Monmouth Junction, NJ, USA).

### 2.2. Duck Embryo Fibroblast (DEF) Primary Cells

Nine-day-old to 11-day-old duck embryos were analyzed for the presence of some specific pathogens, including duck enteritis virus, duck Hepatitis A virus, and DTMUV, before using the embryos to obtain DEF cells. The DEF cells were isolated from specific pathogen-free duck embryos using the trypsin digestion method [21,22] and cultured in complete Dulbecco’s modified eagle’s medium (DMEM) supplemented with 5% fetal bovine serum (FBS, Gibco), and 1% antibiotics (0.1 mg/mL streptomycin and 0.1 mg/mL penicillin) at 37 °C in 5% CO_2_. The experiment with duck embryos has been approved by the Committee of Experiment Operational Guidelines and Animal Welfare of Sichuan Agricultural University, China. The approved permit number is XF2014-18.

### 2.3. Viral Infection

The DTMUV CQW1 strain (GenBank: KM233707.1) used in this study was isolated from a young duck in Southwest China and purified by the plaque method in our laboratory [23]. For DTMUV infection, DEF cells grown to 70–80% confluence in 6-well plates were infected with PBS or DTMUV CQW1 at different multiplicities of infection (MOIs), as specified in the figure legends. After 1 h of absorption, cells were washed once with warm phosphate-buffered saline (PBS) and cultured in 2% FBS culture medium at 37 °C for different times until harvesting.

### 2.4. Plasmids and Small Interfering RNA (siRNA)

The plasmid GFP-LC3 and the tandem fluorescent monomeric red fluorescent protein mRFP-GFP-LC3 (ptfLC3) were gifts from Dr. Yingjie Sun (Shanghai Veterinary Research Institute, Chinese Academy of Agricultural Sciences, China). Plasmid Flag-p62 was generated in pcDNA3.1+ vector and plasmid DsRed-p62 was generated in pDsRed-Expression-N1 vector. The primers for pcDNA3.1-Flag-p62 and pDsRed-p62 are shown in Table 1. Duck-GFP-TBK1, Duck-NFκ-B-luc, Duck-IRF7-luc, and pRL-TK were stored in our laboratory. The siRNAs targeting Beclin 1, LC3B, and p62 were designed and synthesized by GenePharma (Shanghai, China), and the sequences are shown in Table 1. DEF cells were transiently transfected with plasmids or siRNAs using Lipofectamine 3000 (Invitrogen, L3000015, Shanghai, China) following the manufacturer’s instructions.

### 2.5. Transmission Electron Microscopy (TEM)

Samples were fixed in 3% glutaraldehyde and then sent to the Analytical and Testing Center, Sichuan University for TEM observation. Images were obtained with a JSM-7500F transmission electron microscope (JEOL, Tokyo, Japan).

### 2.6. Fluorescence Microscopy

Coverslips were fixed with 4% paraformaldehyde for 30 min at room temperature and mounted by Mounting Medium (Solarbio, S2110, Beijing, China). Then, images were obtained by a Nikon ECLIPSE 80i fluorescence microscope (Nikon, Tokyo, Japan).

### 2.7. Western Blotting

Cells were washed twice in precooled PBS and incubated with radioimmunoprecipitation assay (RIPA) lysis buffer (Solarbio, R0020, Beijing, China) containing 1 mM of phenylmethylsulfonyl fluoride (PMSF, an inhibitor of serine proteases and acetylcholinesterase) (Solarbio, P0100, Beijing, China) at 4 °C for 30 min. Then, the lysates were clarified by centrifugation at 12,000× *g* for 10 min at 4 °C, and the concentration of extractive protein was measured using a bicinchoninic acid (BCA) protein assay kit (Solarbio, PC0020, Beijing, China). Equal amounts of protein samples were boiled for 5 min in 4 × SDS-PAGE loading buffer, separated on 12–15% SDS-PAGE gels, and then electrotransferred onto polyvinylidene fluoride (PVDF) membranes (Bio-Rad, 162-0177, Hercules, CA, USA). Then, the PVDF membranes with the target proteins were blocked for 2 h at room temperature in tris-buffered saline and Tween 20 (TBST) containing 5% nonfat milk powder. After that, the membranes were incubated with primary antibodies (1:1000) at 4 °C overnight; then, with the corresponding secondary antibodies (1:5000), they were conjugated to HRP at 37 °C for 1 h. The protein bands were detected by an ECL Plus kit (Solarbio, PE0010, Beijing, China) and imaged by ChemiDoc MP (Bio-Rad, Hercules, CA, USA). The loading control in this study was β-actin. The gray values of protein blots were measured by the Image Lab software.

### 2.8. Pharmaceutical Treatment

E64d was prepared in DMSO, and cells were treated at the concentration of 10 μg/mL. Rapamycin was prepared in DMSO, and cells were treated at the concentration of 1 μM. In addition, 3-MA was prepared in double-distilled water, and cells were treated at the concentration of 5 mM. Finally, CQ was prepared in double-distilled water, and cells were treated at the concentration of 20 μM.

### 2.9. Quantitative RT-PCR Assay

The mRNA levels of interferon-alpha (IFN-α), interferon-beta (IFN-β), and interleukin 6 (IL-6) were detected by RT-PCR. The total RNA from DEF cells was extracted with RNAiso Plus reagent, and subsequently reverse transcribed into cDNA using a PrimeScript™ RT reagent Kit (Takara, RR047A, Dalian, China) according to the manufacturer’s protocol. Quantitative PCR (qPCR) was performed using the Bio-Rad CFX96 Real-Time Detection System (Bio-Rad, Hercules, CA, USA), and the β-actin was selected as the housekeeping gene. The sequences of the gene-specific primers used for qPCR are shown in Table 1.

### 2.10. Luciferase Reporter Assay

DEF cells grown in 96-well plates were co-transfected with 0.1 μg of NF-κB–luc or IRF7-luc and with 0.01 μg of pRL-TK plasmid, along with the indicated siRNA or plasmids, as specified in the figure legend. After 24 h post-transfection, cells were infected with DTMUV at an MOI of 1 for 24 h. Cell lysates were prepared and analyzed for Firefly and Renilla luciferase activities using the Dual-Glo Luciferase Assay System (Promega, E2920, Madison, WI, USA), following the manufacturer’s instructions.

### 2.11. Median Tissue Culture Infectious Doses (TCID50) Assay

To determine the virus titer of the DTMUV, DEF cells cultivated in 96-well plates were inoculated with 10-fold serial dilutions of samples and incubated at 37 °C for 7 days. The virus titer was calculated on the basis of the Spearman–Kaerber algorithm and expressed TCID50 per 0.1 mL.

### 2.12. Statistical Analysis

Data are presented as means ± standard deviations (SD). The significance of the variability between different treatment groups was analyzed by the two-tailed independent Student *t*-test using the GraphPad Prism software (version 6.0, GraphPad, San Diego, CA, USA). A *p*-value of < 0.05 was considered to be statistically significant.

## 3. Results

### 3.1. DTMUV Infection Induces the Formation of Autophagosome-Like Vesicles in DEF Cells

**Figure 1:**
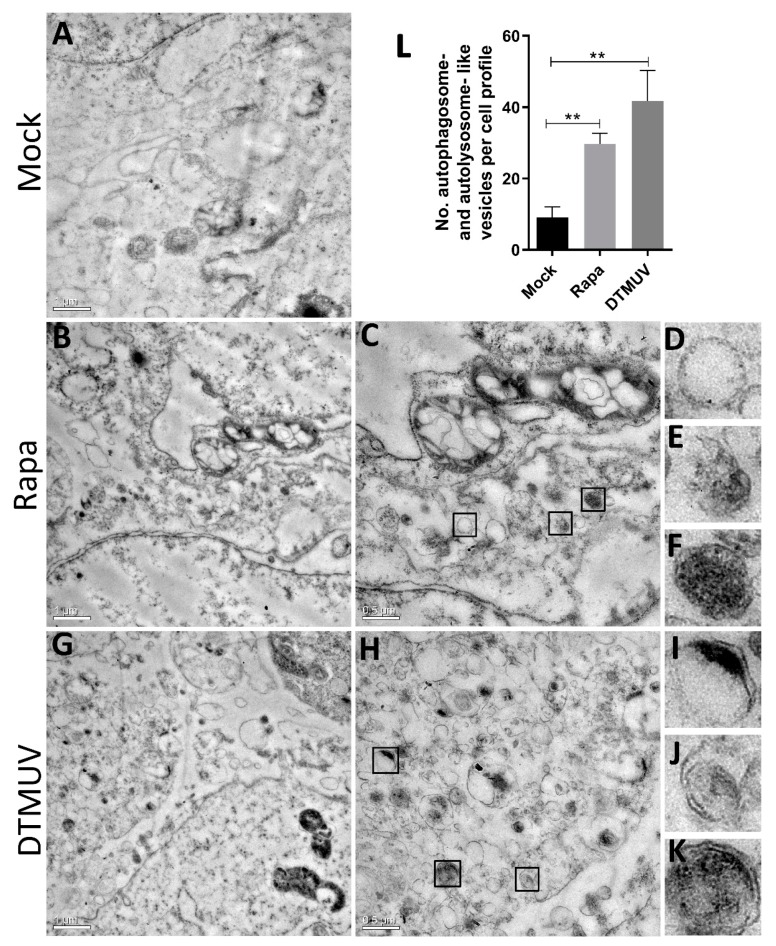
Observation of autophagy-like vesicle formation by TEM. (**A**) Duck embryo fibroblast (DEF) cells were mock-infected as a negative control. (**B**–**F**) DEF cells were treated with rapamycin (Rapa) (1 μM) as a positive control. Panel (**C**) was a higher-magnification view of panel (**B**). Panels (**D**–**F**) were further enlargements of mature autophagosome-like, immature autophagosome-like, and autolysosome-like vesicles, respectively. (**G**–**K**) Duck embryo fibroblast (DEF) cells were infected with DTMUV CQW1 at a multiplicity of infection (MOI) of 1 for 36 h. Panel H was a higher-magnification view of panel (**G**). Panels (**I**–**K**) were further enlargements of immature autophagosome-like, mature autophagosome-like, and autolysosome-like vesicles respectively. (**L**) Quantification of the number of autophagosome-like and autolysosome-like vesicles per cell profile in mock-infected, Rapa-treated, and Duck Tembusu virus (DTMUV)-infected DEF cells. Average number in each cell profile was obtained from 10 cell profiles undergoing each treatment. The data are represented as the mean ± SD from three independent experiments. Two-tailed Student’s *t* test, ** *p* < 0.01.

To determine whether DTMUV infection triggered cellular autophagy, we first examined the formations of autophagosome-like vesicles in DEF cells by TEM. Mammalian target of rapamycin complex 1 (mTORC1) is an autophagy-suppressive regulator that integrates growth factor, nutrient, and energy signals and the inhibition of mTOR, leading to an induction of autophagy in most cells [13]. Rapa, an a potent and specific mTOR inhibitor, has been used as an inducer for autophagy in many mammalian cells [24]. In this study, we use Rapa treatment in DEF cells as a positive control. We found many autophagosome-like vesicles (Figure 1B,C) in Rapa-treated DEF cells but just a few in mock-infected cells. The characteristics of immature autophagosome-like vesicles (Figure 1E), mature autophagosome-like vesicles (Figure 1D), and autolysosome-like vesicles (Figure 1F) were very obvious in cells with Rapa treatment. Furthermore, there were many more autophagosome-like and autolysosome-like vesicles found in DTMUV-infected cells (Figure 1G–K). Quantitative analysis also showed a significant increase in the quantification of autophagosome-like and autolysosome-like vesicles in the DTMUV-infected cells and Rapa-treated cells compared to those in the mock-infected cells (*p* < 0.01) (Figure 1L). We also have taken some images of mock-infected, DTMUV-infected and Rapa-treated DEF cells under a normal microscopy to track DTMUV replication in DEF cells (Figure S1).

### 3.2. DTMUV Infection Increases the Levels of Autophagic Markers in DEF Cells

**Figure 2:**
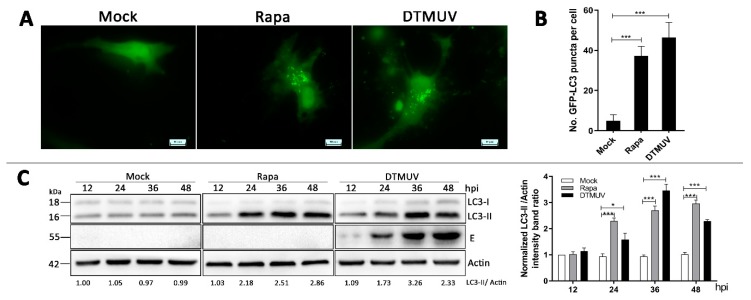
Measurement of autophagic markers in DEF cells infected with DTMUV. (**A**) DEF cells were transfected with GFP-LC3 plasmids for 24 h prior to experiment. Then, cells were mock-treated as a negative control, Rapa treated (1 μM) as a positive control, or infected with DTMUV at an MOI of 1 for 36 h. Cells were fixed and imaged for GFP fluorescence. The images shown were representative of three independent experiments. (**B**) Quantification of the numbers of GFP-LC3 puncta per cell in each group were quantified, and the average number of the puncta in each cell was obtained from 50 cells undergoing each treatment. (**C**) DEF cells were mock-infected, Rapa treated, or infected with DTMUV at an MOI of 1 for 12, 24, 36, and 48 h. Samples were harvested for Western blot analysis and immunoblotted for LC3, DTMUV-E, and β-actin. The ratio of LC3-II to β-actin was normalized to control conditions in mock-infected cells. Error bars: Mean ± SD of three independent experiments. Two-tailed Student’s *t*-test; * *p* < 0.05, *** *p* < 0.001 compared to control.

To further analyze whether the autophagy can be triggered by DTMUV infection in DEF cells, green fluorescent protein-LC3 (GFP-LC3) plasmids were transfected to observe LC3 puncta formation, which indicated the autophagic response. Obvious GFP-LC3 puncta were observed in DTMUV-infected and Rapa-treated DEF cells but rarely in mock-infected cells (Figure 2A). Quantitative analysis also showed a significant increase in the number of LC3 puncta in DTMUV-infected and Rapa-treated DEF cells compared with those in mock-infected cells (*p* < 0.001) (Figure 2B).

We also examined the level of LC3 protein to monitor autophagy process by Western blotting. As shown in Figure 2B, in comparison to mock-infected cells, the amount of LC3-II in DTMUV-infected cells was significantly upregulated from 24 h to 36 h post-infection (hpi) where there was a little drop from 36 hpi to 48 hpi. In Rapa-treated cells, the LC3-II protein level was upregulated continuously from 24 h to 48 h. Meanwhile, the levels of DTMUV envelope protein E were measured to track the procession of infection.

### 3.3. DTMUV Infection Enhances Autophagic Flux in DEF Cells

**Figure 3:**
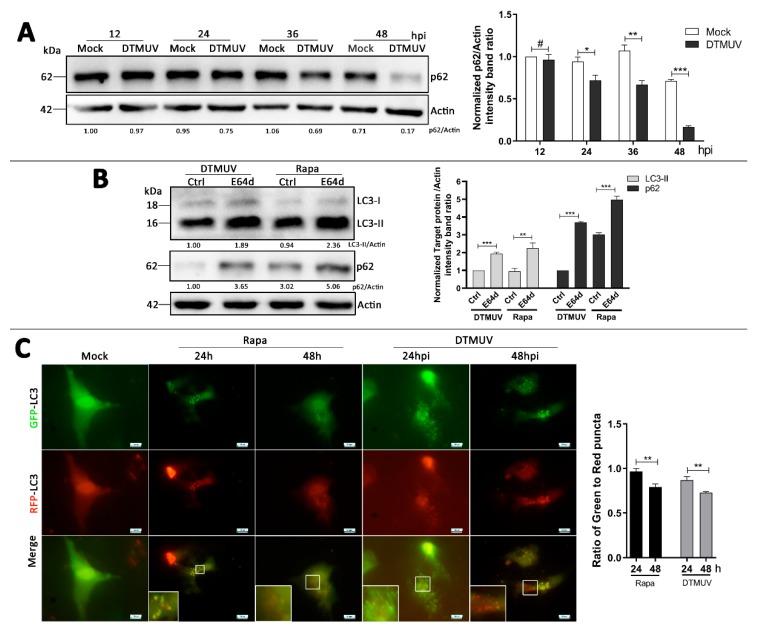
Measurement of the autophagic flux in DTMUV-infected cells. (**A**) DEF cells were mock-infected or infected with DTMUV at an MOI of 1 for 12, 24, 36, and 48 h. Samples were harvested for Western blot analysis and immunoblotted for p62 and β-actin. The ratio of p62 to β-actin was normalized to control conditions in mock-infected cells at 12 h. (**B**) DEF cells were infected with DTMUV CQW1 (MOI = 1) in the presence or absence of E64d (10 μg/mL) for 24 h post-infection (hpi) and 48 hpi. DEF cells were subjected to a 1-h absorption period of DTMUV and further cultured in fresh medium in the absence (Ctrl) or presence (E64d) of E64d (10 µg/mL) for 48 hpi. Rapa-treated cells were used as controls. Samples were harvested for Western blot analysis and immunoblotted for LC3, p62, and β-actin. The ratios of targeting proteins to β-actin were normalized to control conditions in the absence of E64d. (**C**) DEF cells were transfected with ptf-LC3 plasmids for 24 h prior to the experiment. Then, cells were mock-infected, Rapa-treated, or infected with DTMUV at an MOI of 1 for 24 hpi and 48 hpi. Cells were fixed and imaged for the fluorescence of GFP and red fluorescent protein (RFP). Images shown were representative of three independent experiments. Ratio of green to red puncta per cell in each group were quantified and obtained from 50 cells undergoing each treatment. Error bars: Mean ± SD of three independent experiments. Two-tailed Student’s *t* test; # *p >* 0.05, * *p* < 0.05, ** *p* < 0.01, *** *p* < 0.001.

To make an accurate observation of autophagic flux, we used three approaches to determine whether the autophagic flux was activated in DTMUV-infected cells, including p62 degradation, the turnover of LC3-II [25], and the mRFP-GFP-LC3 tandem fluorescent protein quenching assay [26]. Firstly, as shown in Figure 3A, there were no changes of p62 protein level from 12 h to 36 h, but a little drop at 48 h in mock-infected cells. In comparison, DTMUV infection increased the degradation of p62 in DEF cells over time, and p62 levels were lower than those in infected cells all the time. Secondly, we measured the protein levels of LC3-II and p62 in the presence or absence of the lysosome inhibitor E64d in DTMUV-infected cells. E64d is a membrane-permeative inhibitor of the cathepsins B, H, and L and is also widely used in autophagic flux analysis experiments [27]. As shown in Figure 3B, E64d treatment increased the levels of LC3-II and p62 in DTMUV-infected and Rapa-treated cells, respectively, compared to those in the control group. In addition, we transfected the ptfLC3 plasmids, a tandem reporter construct, into DEF cells to monitor the quenching process of LC3 puncta in DTMUV-infected cells. As shown in Figure 3C, DTMUV infection in ptfLC3-transfected DEF cells led to a gradual shift from yellow to red fluorescence and a significant increase of free-red puncta from 24 hpi to 48 hpi. The ratio of green puncta to red puncta was also decreased significantly over time (*p* < 0.01) (Figure 3C), and there was a similar observation in Rapa-treated cells (Figure 3C).

### 3.4. Autophagy-Altering Treatments Affect the Replication of DTMUV in DEF Cells

**Figure 4:**
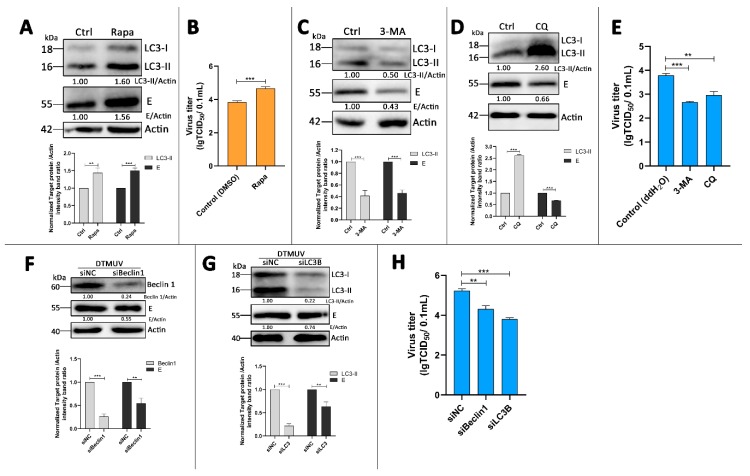
Effect of autophagy on DTMUV replication. (**A**,**C**,**D**) DEF cells were treated with Rapa (500 nM, DMSO as control) (**A**), 3-methyladenine (3-MA) (5 mM, ddH_2_O as control) (**C**), chloroquine (CQ) (20 μM, ddH_2_O as control) (**D**) for 4 h prior to infection and then infected with DTMUV at an MOI of 1 for 24 h. Samples were harvested for Western blot analysis and immunoblotted for LC3, DTMUV-E, and β-actin. The ratios of targeting proteins to β-actin were normalized to control. (**B**,**E**) DEF cells were with the same pharmaceutical treatments as in (**A**,**C**,**D**), and then infected with DTMUV at an MOI of 0.01 for 24 h. Progeny virus yields in DEF cells were determined by TCID50 assay. (**F**,**G**) DEF cells were transfected with siBeclin 1 (**F**), siLC3B (**G**), or siNC for 24 h prior to infection and then infected with DTMUV at an MOI of 1 for 36 h. Samples were harvested for Western blot analysis and immunoblotted for LC3, DTMUV-E, and β-actin. The ratios of targeting proteins to β-actin were normalized to control. (**H**) DEF cells were with the same transfections as in (**F**,**G**), and then infected with DTMUV at an MOI of 0.01 for 36 h. Progeny virus yields in DEF cells were determined by TCID50 assay. Error bars: Mean ± SD of three independent experiments. Two-tailed Student’s *t*-test; ** *p* < 0.01, *** *p* < 0.001.

To analyze the effect of autophagy on DTMUV replication, we treated cells with Rapa, which is an autophagy inducer that was described above. We found that Rapa treatment enhanced the protein levels of LC3-II and DTMUV-E in DTMUV-infected cells (Figure 4A) and increased the viral progeny titer significantly compared to that in the control group (Figure 4B).

As autophagy activation enhanced DTMUV replication, we analyzed the effect of autophagy inhibition next. An autophagy inhibitor, 3-MA, inhibits the activity of phosphatidylinositol 3-kinase (PI3K) to prevent the formation of autophagosomes. Figure 4C showed that 3-MA treatment reduced the protein levels of LC3-II and DTMUV-E, and the viral titers of DTMUV progeny were also significantly reduced in DEF cells (Figure 4E). Another autophagy inhibitor, CQ, raises the lysosomal pH to inhibit the fusion of autophagosome with lysosome and lysosomal protein degradation [28]. The results showed that CQ treatment caused a large accumulation of LC3-II and reduced E protein levels (Figure 4D), and the results of viral progeny titers were similar with 3-MA-treated cells (Figure 4E).

To eliminate problems associated with pharmaceutical autophagy regulators and further confirm these results, we used siRNA transfection experiments to knockdown endogenous Beclin1 and LC3B proteins. Beclin1 and LC3B proteins are both encoded by autophagy-related genes (ATGs) and play essential roles in the signaling pathways involved in the formation of autophagosomes. The results showed that DEF cells transfected with siBeclin1 or siLC3B had a significant decrease in endogenous Beclin 1 or LC3B proteins respectively compared with those in no-targeting siRNAs (siNC)-transfected cells (Figure 4F,G). Moreover, the knockdown of Beclin 1 and LC3B resulted in the decrease of E protein expression (Figure 4F,G) and the viral progeny titers in DTMUV-infected cells (Figure 4H). These results suggested that the autophagy process was required for effective infection by DTMUV.

### 3.5. Absence of Autophagy Enhances Type I Interferons and IL-6 Production in DTMUV-Infected Cells

**Figure 5:**
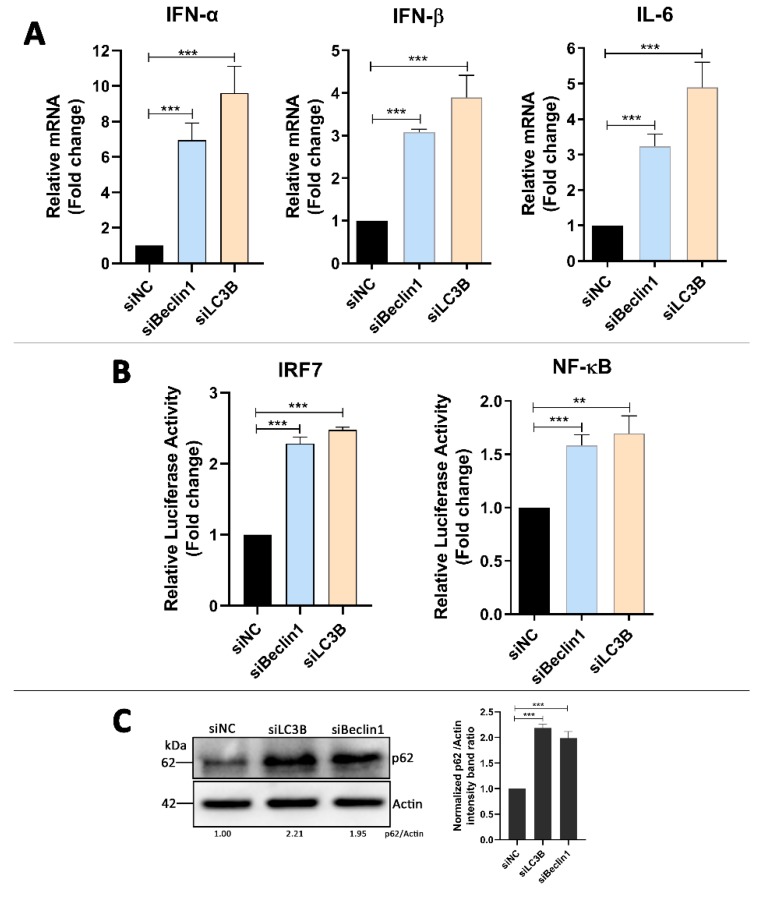
Effect of autophagy on innate immune responses in DTMUV-infected cells. (**A**) DEF cells were transfected with siNC, siBeclin 1, or siLC3B for 24 h prior to infection and then infected with DTMUV at an MOI of 1 for 36 hpi. qPCR analysis were performed for the mRNA levels of IFN-α, IFN-β, and IL-6. The mRNA levels of targeting genes to β-actin were normalized to control. (**B**) DEF cells were co-transfected with 0.1 μg of NF-κB-luc or IRF7-luc and with 0.01 μg of the HSV-thymidine kinase promoter (pRL-TK) plasmid, along with siNC, siBeclin 1, or siLC3B for 24 h prior to infection. Cells were infected with DTMUV at an MOI of 1 for 36 hpi and then preformed for luciferase reporter assays. The luciferase activities of the targeting promoters were normalized to control. (**C**) Parallel samples from (**A**) were analyzed by Western blot and immunoblotted for LC3, DTMUV-E, and β-actin. The ratio of p62 to β-actin was normalized to control. Error bars: Mean ± SD of three independent experiments. Two-tailed Student’s *t* test; ** *p* < 0.01, *** *p* < 0.001.

Based on the above results, we wanted to know how the autophagy attenuation inhibited DTMUV replication in DEF cells. DEF cells were transfected with siBeclin 1 or siLC3B for 24 h prior to infection and then infected with DTMUV for 36 hpi. Cells were harvested for testing mRNA levels of IFN-α, IFN-β, and IL-6 by qPCR. As shown in Figure 5A, the knockdown of Beclin 1 or LC3B enhanced the expression of IFN-α, IFN-β, and IL-6 significantly compared to those in siNC-transfected cells. To test the effects on the upstream type I interferons and IL-6 in Beclin 1 or LC3B knockdown cells, DEF cells were co-transfected with the IRF7 or NF-kB luciferase reporter plasmids, along with siBeclin1 or siLC3B for 24 h, after which they were infected with DTMUV for 36 hpi. The data showed that the knockdown of Beclin 1 or LC3B enhanced the activation of the IRF7 and NF-kB promoter compared to those in siNC-transfected cells (Figure 5B). In the parallel samples of mRNA level testing experiments, we found that p62 protein levels were enhanced in siBeclin1 or siLC3B knockdown cells, which meant that the degradation of p62 by autolysosomes was inhibited (Figure 5C). Thus, we have a hypothesis that p62 plays a key role in the innate immune responses induced by DTMUV infection. The mRNA levels of IFN-α, IFN-β, and IL-6 in mock-infected or only DTMUV-infected DEF cells also have been tested (Figure S2).

### 3.6. p62 Regulates IRF7 and NF-kB Pathways and DTMUV Replication in DTMUV-Regulated Cells

**Figure 6:**
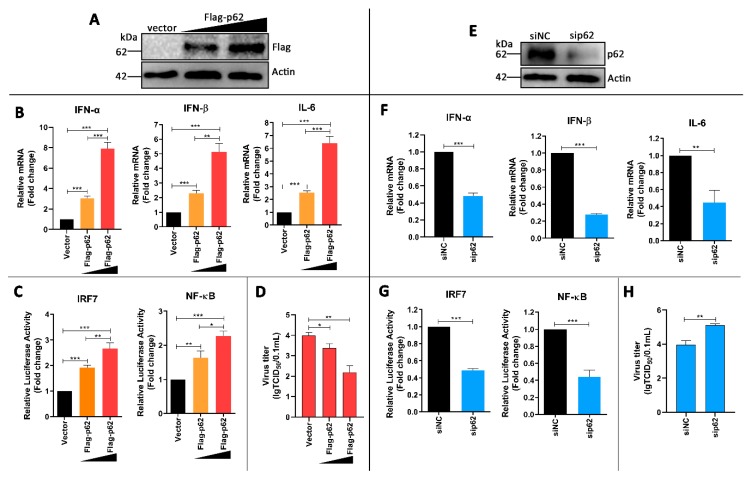
Effect of p62 on innate immune responses and DTMUV replication in DTMUV-infected cells. (**A**) DEF cells were transfected with vector, Flag-p62 (1 μg), or Flag-p62 (2 μg) plasmids for 24 h prior to infection and then infected with DTMUV at an MOI of 1 for 24 hpi. Cells were harvested for Western blot analysis and immunoblotted for Flag and β-actin. (**B**) Parallel samples from (**A**) were performed for the mRNA levels of IFN-α, IFN-β, and IL-6 by qPCR. The mRNA levels of targeting genes to β-actin were normalized to control. (**C**) DEF cells were co-transfected with 0.1 μg of NF-κB–luc or IRF7-luc and with 0.01 μg of pRL-TK plasmid, along with vector, Flag-p62 (1 μg) or Flag-p62 (2 μg) plasmids for 24 h prior to infection. Cells were infected with DTMUV at an MOI of 1 for 36 h and then preformed for luciferase reporter assays. The luciferase activities of targeting promoters were normalized to control. (**D**) Progeny virus yields in parallel samples from (**A**) were determined by TCID50 assay. (**E**) DEF cells were transfected with siNC or sip62 for 24 h prior to infection and then infected with DTMUV at an MOI of 1 for 24 h. Cells were harvested for Western blot analysis and immunoblotted for p62 and β-actin. (**F**) Parallel samples from (**E**) were performed for the mRNA levels of IFN-α, IFN-β, and IL-6 by qPCR. The mRNA levels of targeting genes to β-actin were normalized to control. (**G**) DEF cells were co-transfected with 0.1 μg of NF-κB–luc or IRF7-luc and with 0.01 μg of pRL-TK plasmid, along with siNC or sip62 for 24 h prior to infection. Cells were infected with DTMUV at an MOI of 1 for 24 hpi and then preformed for luciferase reporter assays. The luciferase activities of targeting promoters were normalized to control. (**H**) Progeny virus yields in parallel samples from (**E**) were determined by TCID50 assay. Error bars: Mean ± SD of three independent experiments. Two-tailed Student’s *t*-test; * *p* < 0.05, ** *p* < 0.01, *** *p* < 0.001.

To test our hypothesis, DEF cells were transfected with Flag-p62 plasmids for 24 h and then infected with DTMUV for 24 hpi. We found that Flag-p62 plasmids were expressed successfully in DEF cells (Figure 6A) and p62 replenishment increased the mRNA levels of IFN-α, IFN-β, and IL-6 compared to those in vector-transfected cells (Figure 6B). The luciferase reporter experiments also showed that p62 replenishment enhanced the activation of IRF7 and NF-kB promoters (Figure 6C). We further found that p62 replenishment inhibited DTMUV replication significantly. Moreover, all the changes were associated with the amount of Flag-p62 transfection and in a dose-dependent manner.

To further confirm the above results, the effects of knockdown were analyzed by using sip62. As shown in Figure 6E, p62 expression was successfully knocked down in sip62-transfected cells. Further, p62 depletion decreased the mRNA levels of IFN-α, IFN-β, and IL-6 (Figure 6F), and it also inhibited the activation of IRF7 and NF-kB promoters (Figure 6G). Furthermore, p62 depletion increased DTMUV replication significantly (Figure 6H). Altogether, these results confirm our hypothesis that p62 plays a key role in in regulating DTMUV-induced innate immune responses and DTMUV replication.

### 3.7. p62 Regulates the Phosphorylation of TBK1 (p-TBK1) in DTMUV-Infected Cells

**Figure 7:**
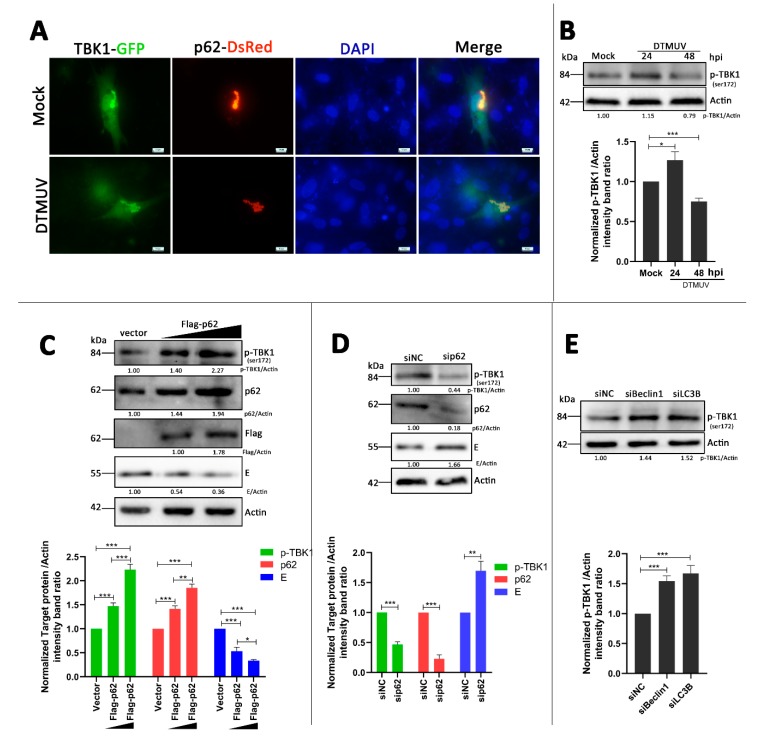
TBK1 is a target of p62 modulating innate immune responses. (**A**) DEF cells were co-transfected with GFP-TBK1 and DsRed-p62 plasmids for 24 h prior to experiment. Cells were then mock-infected or infected with DTMUV at an MOI of 1 for 24 hpi. Cells were fixed and imaged for GFP and DsRed fluorescence. Images shown were representative of three independent experiments. (**B**) DEF cells were mock-infected or infected with DTMUV at an MOI of 1 for 24 and 48 h. Samples were harvested for Western blot analysis and immunoblotted for p-TBK1 and β-actin. The ratio of p-TBK1 to β-actin was normalized to control conditions in mock-infected cells. (**C**) DEF cells were treated as described in Figure 6A, and cells were harvested for Western blot analysis and immunoblotted for p-TBK1, p62, Flag, DTMUV-E, and β-actin. The ratios of targeting proteins to β-actin were normalized to control. (**D**) DEF cells were treated as described in Figure 6E. Then, cells were harvested for Western blot analysis and immunoblotted for p-TBK1, p62, DTMMUV-E, and β-actin. The ratios of targeting proteins to β-actin were normalized to control. (**E**) DEF cells were treated as described in Figure 5C. Then, cells were harvested for Western blot analysis and immunoblotted for phosphorylated TANK-binding kinase 1 (p-TBK1) and β-actin. The ratios of targeting proteins to β-actin were normalized to control. Error bars: Mean ± SD of three independent experiments. Two-tailed Student’s *t* test; * *p* < 0.05, ** *p* < 0.01, *** *p* < 0.001.

TANK-binding kinase 1 (TBK1) has been reported to be in the upstream of both the IRF7 pathway [29] and the NF-kB pathway [30] in virus infection. It also has a major role in autophagy [31], and there is colocalization between TBK1 and autophagic cargo adaptors, including p62 [32] and optineurin [33]. Thus, we hypothesized that TBK1 played a role in the p62-regulated IRF7 and NF-kB pathways. To investigate our hypothesis, DEF cells were co-transfected with TBK1-GFP and p62-DsRed plasmids for 24 h and then infected with DTMUV or not (Mock). We found that there was colocalization between TBK1 and p62, and the colocalization occurred both in mock-infected and DTMUV-infected cells (Figure 7A). More cell images were taken to verify this result (Figure S3). We further tested the levels of p-TBK1 in mock-infected and DTMUV-infected cells in DEF cells without exogenous protein expression. As shown in Figure 7B, the levels of p-TBK1 in DTMUV-infected cells were increased at 24 hpi but decreased at 48 hpi compared with those in mock cells. Then, the levels of phosphorylated TBK1 (p-TBK1) were also tested in DEF cells that were transfected with Flag-p62 plasmids or sip62 for 24 h and then infected with DTMUV for 24 h. As shown in Figure 7C,E, p62 replenishment significantly increased the protein level of p-TBK1 compared with that in vector-transfected cells, and the change was in a dose-dependent manner. Furthermore, the level of DTMUV E was decreased, which was consistent with the result in Figure 6E, whereas in p62 knockdown cells, the protein level of p-TBK1 was decreased significantly compared to that in siNC-transfected cells, and the level of DTMUV E was increased, which was consistent with the result in Figure 6H. Flag and p62 bands were measured to confirm the overexpression or the knockdown of p62 in DEF cells. In addition, we found an increase of p-TBK1 level in siBeclin 1 or siLC3B knockdown DEF cells with DTMUV infection (Figure 7E).

## 4. Discussion

Autophagy, an intrinsic process associated with membrane trafficking in eukaryotic cells, is a host response to pathogen infection. In this paper, we first identified that DTMUV infection triggered autophagy in DEF cells. There were multiple hallmarks for activated autophagy. We observed double-membrane vesicles in DTMUV-infected cells and Rapa-treated cells under electron microscopy (Figure 1). In addition, GFP-LC3 puncta formation (Figure 2A) and LC3 conversion (Figure 2B) were observed in DEF cells with DTMUV infection and Rapa treatment. The cell model used in this study, DEF cells, is a primary cell from the original host species of DTMUV, which offers a more realistic host response compared to cell lines. Meanwhile, DEF cells are the only cell model used from duck species so far. To make a more comprehensive understanding of DTMUV-triggered autophagy and the role of autophagy in the life cycle of DTMUV, we may use some other cell lines, such as HEK293 or BHK21 cells, which have been reported to be permissive for DTMUV infection [34,35], to study the interaction between DTMUV replication and autophagy in the future. Autophagy responses occurs in many other flavivirus members, including Hepatitis C virus (HCV) [36,37], JEV [38], dengue virus (DENV) [39], classical swine fever virus (CSFV) [40], and ZIKA [41]. Autophagy responses may be a common reaction in flavivirus infection. However, different flaviviruses activate autophagy through different mechanisms. For example, CSFV induces autophagy by its nonstructural protein 5A (NS5A) [40] and ZIKA by its NS4A and NS4B proteins [41]. DTMUV is the only flavivirus to infect poultry. So, studying the mechanism of DTMUV-triggered autophagy may help us to expand our understanding on flavivirus–host interactions and may give some ideas to other flavivirus-related researches.

Complete autophagy is due not only to an increase of autophagosome formation but also to the degradation substances in the autophagosomes by the lysosomes [13]. We applied three approaches to provide strong evidence that DTMUV infection enhanced autophagic flux in DEF cells (Figure 2), and we also found autolysosome-like vesicles by TEM (Figure 1K), which were a sign of autophagic flux. The downregulation of LC3-II from 36 hpi to 48 hpi in DTMUV-infected cells (Figure 2C) provided further evidence for autophagic flux. CSFV has been shown to trigger autophagic flux [40]. In contrast, some other flavivirus members, such as HCV [42], inhibit autophagic flux to enhance their replication. HCV could also use the autophagic membranes for the assembly of its RNA replication [43], but the function and mechanism of complete autophagy on flavivirus replication are still unknown. Therefore, it is worth studying the mechanism of DTMUV-altering autophagic flux and how DTMUV utilizes autophagic flux for their replication.

Some flaviviruses have been shown to benefit from autophagy, including HCV [37], DENV [44], and ZIKA [45], whereas JEV replication is inhibited by autophagy [46], and WNV replication is independent of autophagy [47]. These findings indicate that autophagy plays multiple roles in modulating flavivirus replication, which may be due to the host specificity and different pathogenic mechanisms of infections [40]. Here, to investigate the role of autophagy in DTMUV replication, we utilized pharmacological treatments and genetic knockdown to modulate autophagic signaling, and then analyzed the level of DTMUV. We found that inducing autophagy with Rapa treatment not only upregulated the protein level of viral E (Figure 4A) but also increased the yield of DTMUV progeny (Figure 4B). Conversely, inhibiting autophagosome formation with 3-MA treatment and autophagic flux with CQ treatment downregulated the protein level of viral E (Figure 4C) and decreased the yield of DTMUV progeny (Figure 4D). Inhibiting autophagy by the knockdown of essential autophagy Belin 1 or LC3B had similar results (Figure 4G,H). These findings indicated that autophagy, including autophagic flux, plays a positive role in DTMUV replication.

In general, autophagy affects flaviviruses replication in two ways. One is to be involved in the assembly and release of the virus by its membrane trafficking character. The other is to adjust the host antiviral immune response [48]. In this study, we found that autophagy inhibition with knockdown of Beclin 1 and LC3B increased the mRNA levels of Type I interferons and IL-6 (Figure 5A) and also enhanced IRF7 and NF-κB promoter activation (Figure 5B). Previous studies have shown that IRF3 is absent in ducks [49], and duck IRF7 plays the role of triggering type I interferon production [50]. Thus, we analyzed the IRF7 promoter activation in this study. This finding is similar to the immune responses caused by HCV infection and JEV infection in cells with autophagy inhibition [37,38,51]. We also noted that the changes of the immune responses caused by siLC3B transfection were more significant than those caused by siBeclin1 transfection, and these changes were consistent with the protein level of SQSTM1/p62 (Figure 5C).

Therefore, we thought that SQSTM1/p62 played a key role in autophagy inhibition-mediated immune response changes. Then, we found that p62 overexpression enhanced IRF7 and NF-κB pathway activation in DTMUV-infected cells (Figure 6B,C) and inhibited DTMUV replication (Figure 6D and Figure 7B), whereas the knockdown of p62 led to the opposite results (Figure 6F–H and Figure 7C). These results indicated that p62 played a positive role in antiviral immune responses and a negative role in DTMUV replication. Recent reports show that p62 acts as a regulator of host immunity by its function as a cargo adaptor in selective autophagy process [52,53,54], whereas SQSTM1/p62 not only functions as a cargo adaptor in the autophagy pathway but also acts as a signaling hub that regulates various physiological processes such as NF-κB signal and antioxidant stress [19,55,56]. Our findings provided evidence of the function of p62 as a regulator of antiviral immune responses and indicated that p62 regulated not only the NF-κB signal but also the IRF7 signal. A recent paper also shows that p62 knockdown enhances innate immune responses and facilitates herpes simplex virus 1 (HSV-1) replication in HSV-1-infected cells [57]. These findings indicated that p62 was a potential antiviral target.

To further investigate how p62 regulates both the NF-κB pathway and the IRF7 pathway, we suspected that p62 played a role in the common upstream of the two pathways. TBK1 has been reported to play a pivotal role in antiviral innate immunity by activating the NF-κB pathway and the IRF3 pathway [58]. It has also been shown to interact with p62 and increase the phosphorylation of p62 [32,59], but the effect of p62 on TBK1 has never been reported. Here, we found full colocalization between p62-RFP and TBK1-GFP in mock-infected or DTMUV-infected cells, but there were no significant changes of the levels of p62 and TBK1 (Figure 7A). We thought it might be due to the exogenous p62 and TBK1 proteins, so, we further tested the levels of p-TBK1 in mock-infected and DTMUV-infected cells in DEF cells without exogenous protein expression (Figure 7B). The level changes of p-TBK1 from 24 to 48 hpi were consistent with those of p62 (Figure 3A), which indicated that there might be some correlation between p62 and p-TBK1. We also found that the level of p-TBK1 was increased with p62 overexpression (Figure 7C), whereas it decreased with p62 knockdown (Figure 7D). In siLC3B- or siBeclin1-transfected cells, the phosphorylation level of TBK1 has also been enhanced (Figure 7E). These findings indicated that p62 functioned as a positive role in antiviral immune response by facilitating the phosphorylation of TBK1. However, the phosphorylation of TBK1 at ser172 is via transautophosphorylation [60]. Therefore, we suspect that p62 plays a role in the dephosphorylation of p-TBK1, which needs to be studied further.

## 5. Conclusions

**Figure 8:**
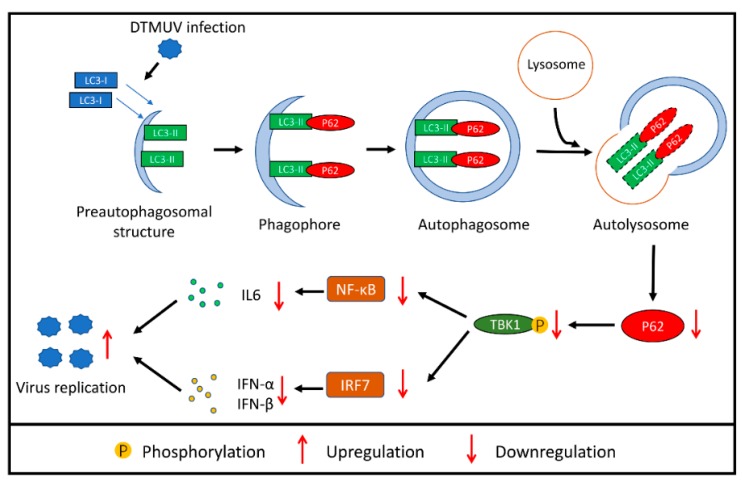
Proposed model of autophagy promotes DTMUV replication by suppressing p62/SQSTM1-mediated innate immune responses in DEF cells.

In summary, these data demonstrate that autophagy promotes DTMUV replication by increasing the degradation of p62 in DEF cells, and one possible mechanism is that p62 degradation inhibits antiviral immune response activation by decreasing the phosphorylation of TBK1 (Figure 8). It is a new strategy for DTMUV to evade host innate immunity. Our study provided a novel insight into DTMUV–host interactions and might provide some basis for the development of new antiviral vaccines and drugs in the future.

## Figures and Tables

**Table 1:** Primer sequences used in this study.

Prime Name	Prime Sequence (5′–3′)	Purpose
Duck-Flag-p62-F	CCGGAATTCATGGATTACAAGGATGACGACGATAAGGCGTTCTCCAGTGA	Gene cloning
Duck-Flag-p62-R	CCGCTCGAGAATACATGTGAGGAGGCTG	
Duck-DsRed-p62-F	CCGGAATTCATGGCGTTCTCCAGTGACG	Gene cloning
Duck-DsRed-p62-R	CGGGGTACCGACATGTGAGGAGGCTG	
Duck-siLC3B-F	GGAGCGCAACCUUCCGUUUTT	Gene Knockdown
Duck-siLC3B-R	AAACGGAAGGUUGCGCUCCTT	
Duck-siBeclin1-F	GCUCAGUACCAGAAGGAAUTT	Gene Knockdown
Duck-siBeclin1-R	AUUCCUUCUGGUACUGAGCTT	
Duck-sip62-F	GCUGCGGAAGAAGCUUCUATT	Gene Knockdown
Duck-sip62-R	UAGAAGCUUCUUCCGCAGCTT	
Duck-IFN-α-F	TCCTCCAACACCTCTTCGAC	RT-qPCR
Duck-IFN-α-R	GGGCTGTAGGTGTGGTTCTG	
Duck-IFN-β-F	AGATGGCTCCCAGCTCTACA	RT-qPCR
Duck-IFN-β-R	AGTGGTTGAGCTGGTTGAGG	
Duck-IL-6-F	TTCGACGAGGAGAAATGCTT	RT-qPCR
Duck-IL-6-R	CCTTATCGTCGTTGCCAGAT	
Duck-β-actin-F	GGTATCGGCAGCAGTCTTA	RT-qPCR
Duck-β-actin R	TTCACAGAGGCGAGTAACTT	

## Supplementary Materials

The following are available online at https://www.mdpi.com/2076-393X/8/1/22/s1, Figure S1: DEF cells were mock infected, Rapa treated or infected with DTMUV at an MOI of 1 for 48 h. These photos were taken under a normal microscopy. Figure S2: DEF cells were mock-infected, DTMUV-infected, transfected with siNC, siBeclin1 or siLC3B for 24 h prior to infection and then infected with DTMUV at an MOI of 1 for 36 hpi. qPCR analysis was performed for the mRNA levels of IFN-α, IFN-β and IL-6. The mRNA levels of targeting genes to β-actin were normalized to control. Figure S3: More cell images of the experiment in Figure 7A. Figure S4: The original blot figures of Figure 2C. (A) The bands of LC3-I/II with weight markers. (B) The bands of DTMUV-E with weight markers. (C) The bands of Actin with weight markers. Figure S5: (A) The original blot figures of Figure 3A. And The bands of p62 and Actin with weight markers. (B) The original blot figures of Figure 3B. And The bands of LC3-I/II, p62 and Actin with weight markers. Figure S6: The original blot figures of Figure 5C. And The bands of p62 and Actin with weight markers. Figure S7: The original blot figures of Figure 6A. And The bands of Flag and Actin with weight markers. Figure S8: The original blot figures of Figure 7E. And The bands of p-TBK1 and Actin with weight markers.
