# Supplementary material for: Autophagy Promotes Duck Tembusu Virus Replication by Suppressing p62/SQSTM1-Mediated Innate Immune Responses In Vitro
Source: Vaccines (Basel). 2020 Jan 13;8(1):22. doi: 10.3390/vaccines8010022 (PMC7157248; doi:10.3390/vaccines8010022)
Supplement: Supplementary file 1 [file vaccines-08-00022-s001.pdf]

vaccines

Supplemental Material

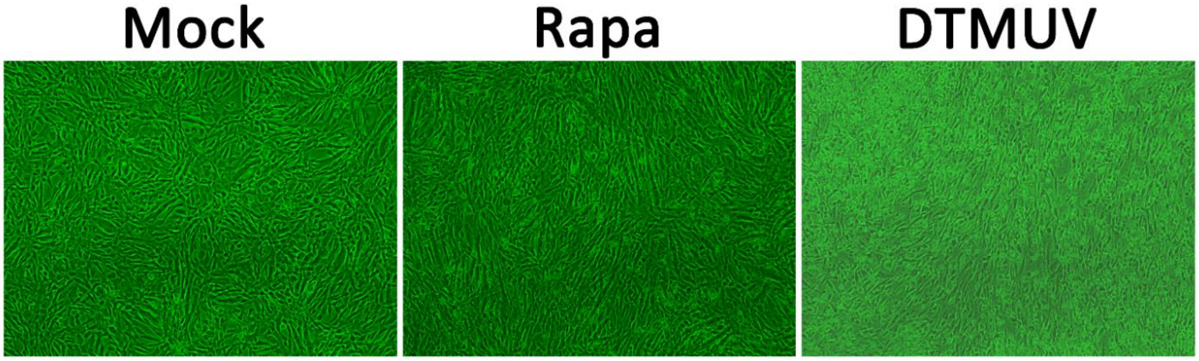

Figure S1. DEF cells were mock infected, Rapa treated or infected with DTMUV at an MOI of 1 for 48h. These photos were taken under a normal microscopy.

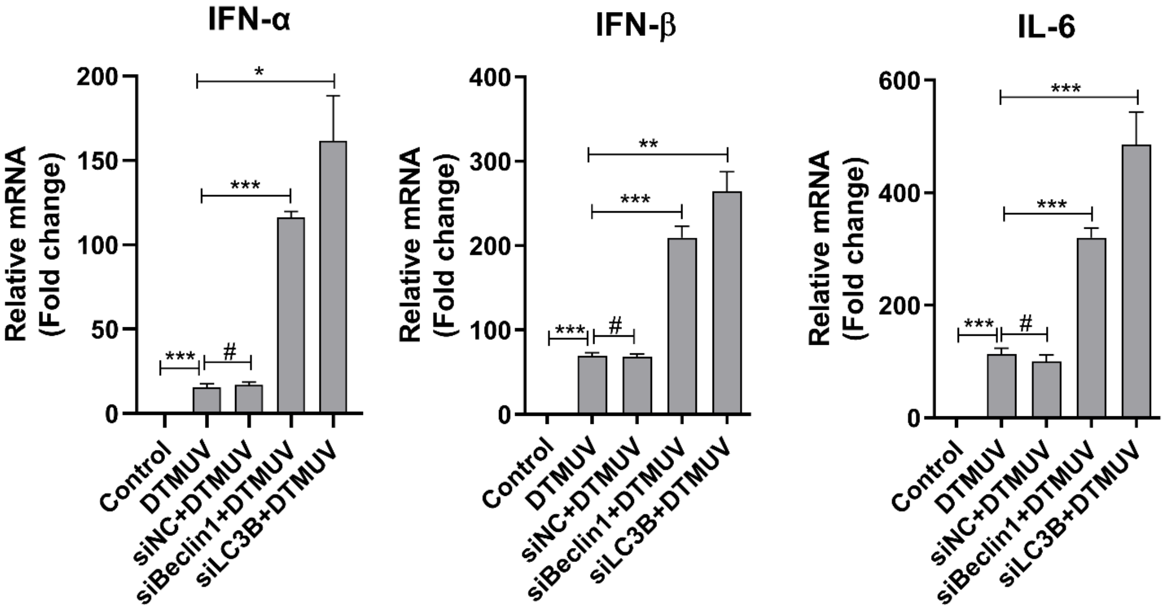

Figure S2. DEF cells were mock-infected, DTMUV-infected, transfected with siNC, siBeclin1 or siLC3B for 24 h prior to infection and then infected with DTMUV at an MOI of 1 for 36hpi. qPCR analysis was performed for the mRNA levels of IFN-α, IFN-β and IL-6. The mRNA levels of targeting genes to β-actin were normalized to control.

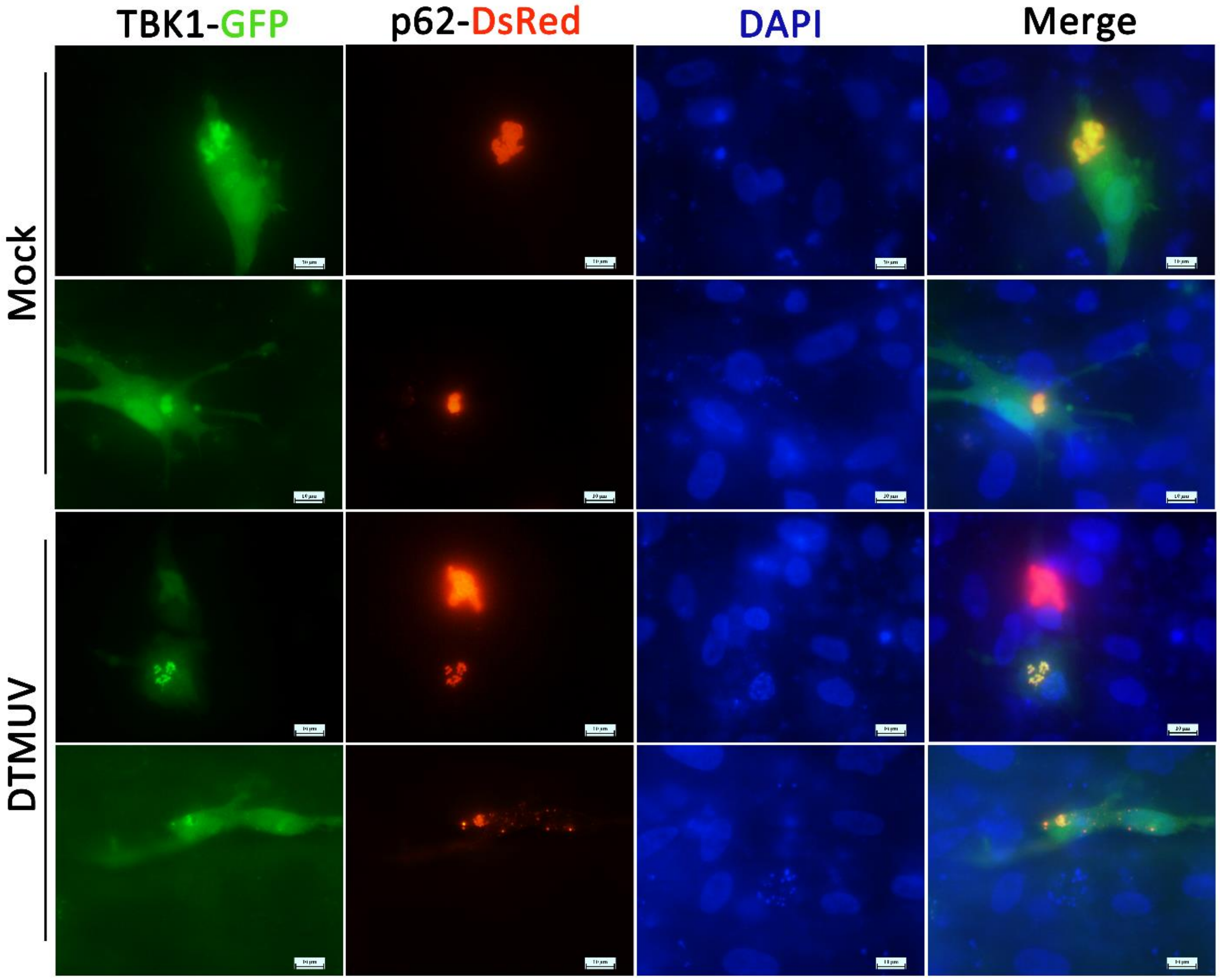

Figure S3. More cell images of the experiment in Figure 7A.

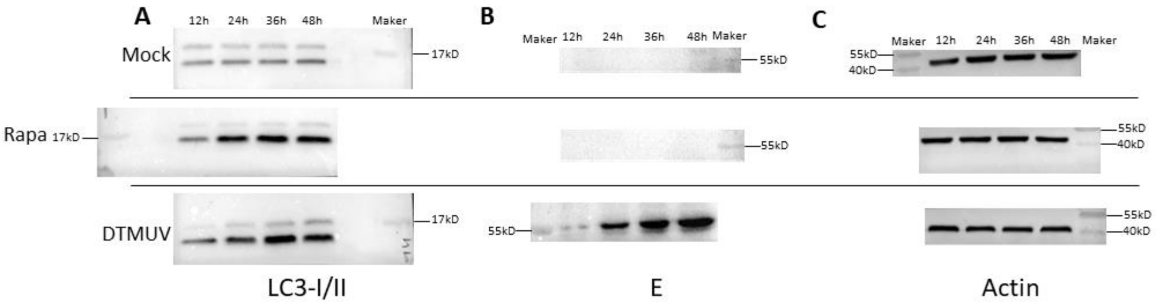

Figure S4. The original blot figures of Figure **2C**. (**A**) The bands of LC3-I/II with weight markers. (**B**) The bands of DTMUV-E with weight markers. (**C**) The bands of Actin with weight markers.

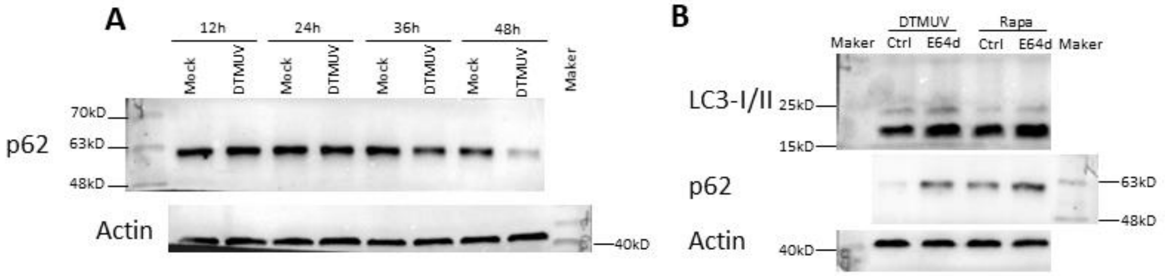

Figure S5. (**A**) The original blot figures of Figure **3A**. And The bands of p62 and Actin with weight markers. (**B**) The original blot figures of Figure **3B**. And The bands of LC3-I/II, p62 and Actin with weight markers.

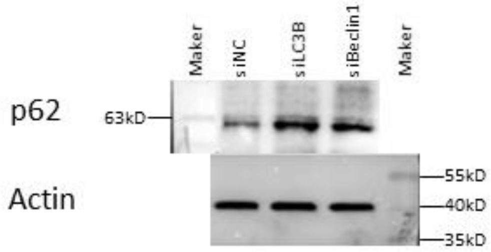

Figure S6. The original blot figures of Figure **5C**. And The bands of p62 and Actin with weight markers.

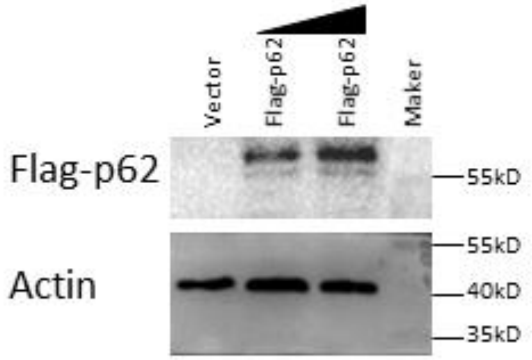

Figure S7. The original blot figures of Figure **6A**. And The bands of Flag and Actin with weight markers.

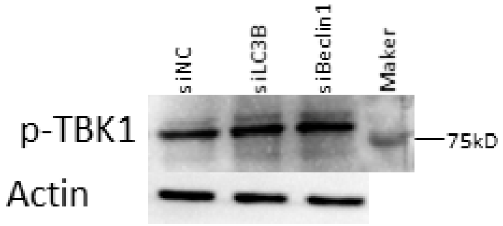

Figure S8. The original blot figures of Figure **7E**. And The bands of p-TBK1 and Actin with weight markers.

© 2020 by the authors. Submitted for possible open access publication under the terms and conditions of the Creative Commons Attribution (CC BY) license (http://creativecommons.org/licenses/by/4.0/).
